# Assessing Native Liver Post-Kasai Portoenterostomy for Biliary Atresia Through Stool Proteome Analysis

**DOI:** 10.1016/j.gastha.2025.100688

**Published:** 2025-04-29

**Authors:** Eiichiro Watanabe, Takeshi Saito, Masahito Yoshihara, Ryo Konno, Jun Fujishiro, Shinya Takazawa, Akinori Ichinose, Kazue Miyake, Tomo Kakihara, Tetsuya Ishimaru, Akira Nishi, Akinari Fukuda, Mureo Kasahara, Osamu Ohara, Yusuke Kawashima

**Affiliations:** 1 Faculty of Medicine, Department of Pediatric Surgery, The University of Tokyo, Bunkyo-ku, Tokyo, Japan; 2 Division of Surgery, Department of Surgical Specialties, National Center for Child Health and Development, Setagaya-ku, Tokyo, Japan; 3 Department of General-Pediatric Hepatobiliary Pancreatic Surgery, Kitasato University School of Medicine, Sagamihara, Kanagawa, Japan; 4 Institute for Advanced Academic Research (IAAR), Chiba University, Chiba, Japan; 5 Department of Artificial Intelligence Medicine, Graduate School of Medicine, Chiba University, Chiba, Japan; 6 Premium Research Institute for Human Metaverse Medicine (WPI-PRIMe), Osaka University, Suita, Osaka, Japan; 7 Department of Applied Genomics, Kazusa DNA Research Institute, Kisarazu, Chiba, Japan; 8 Department of Surgery, Gunma Children’s Medical Center, Shibukawa, Gunma, Japan; 9 Organ Transplantation Center, National Center for Child Health and Development, Setagaya-ku, Tokyo, Japan

**Keywords:** Biliary Atresia, Kasai Portoenterostomy, Stool Proteome Analysis, Cholangitis, C-Reactive Protein

## Abstract

**Background and Aims:**

Biliary atresia (BA) is a severe neonatal condition, characterized by jaundice and hyperbilirubinemia, resulting in cholestasis. Although early diagnosis followed by Kasai portoenterostomy (KPE) can rescue patients, they are prone to complications such as cholangitis. Moreover, a comprehensive study assessing intestinal environment is currently lacking. Therefore, in this study, we aimed to elucidate the stool protein profiles of patients with BA following KPE, provide insights into the native liver condition of BA, and open new avenues for clinical approaches through stool proteome analysis.

**Methods:**

In this prospective study, stool proteome analysis was conducted on samples from 4 patients with well-controlled conditions, 4 patients with repeated cholangitis, and 3 patients with prolonged jaundice without cholangitis, all of whom had undergone KPE. Fifteen healthy individuals without BA were included for comparison.

**Results:**

Principal component analysis revealed that the stool profiles of patients post-KPE with favorable outcomes closely resembled those of healthy controls, whereas the profiles of patients with unfavorable outcomes showed distinct patterns. Notably, C-reactive protein levels were elevated, whereas sodium/hydrogen exchanger 3 levels were decreased in the group with repeated cholangitis.

**Conclusion:**

This study highlights distinct differences in stool protein profiles following KPE, particularly in patients with poor clinical outcomes. This suggests that stool proteome analysis has the potential to provide insights into the native liver conditions of BA patients post-KPE, reflecting their clinical status.

## Graphical abstract

**Figure:**
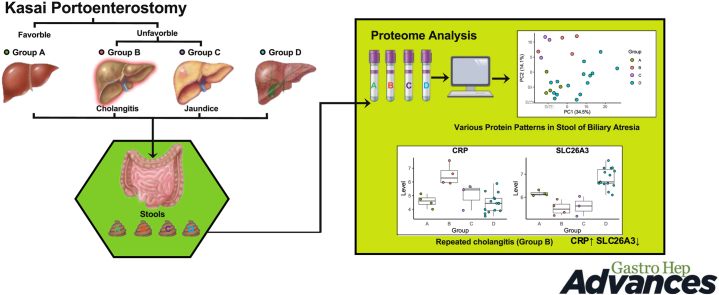

## Introduction

Biliary atresia (BA) is a severe neonatal condition, characterized by jaundice and hyperbilirubinemia that damages various parts of the bile duct, leading to cholestasis.^1^ Early diagnosis, followed by Kasai portoenterostomy (KPE), is crucial for rescuing patients with BA; however, complications such as recurrent cholangitis after KPE progressively harm the native liver, affect the patient's quality of life, and often necessitate early consideration for liver transplantation.^2^, ^3^, ^4^ Therefore, post-KPE assessment of the intestinal environment is essential to provide appropriate treatment, if needed. A potential solution in this regard lies in deep proteomic analysis of stools, which offers insights into the intestinal environment.^5^ A previous study using this method has highlighted differences in host-derived stool protein composition between pre-KPE BA and infantile cholestasis.^6^ Moreover, stools contain abundant proteins from gastrointestinal tissues and have various functions, including in the hepatobiliary tract.^6^^,^^7^ Thus, extensive examination of stool proteins through deep proteome analysis could reveal the obscured intestinal environment, aiding in understanding hepatic pathophysiology in this pediatric population.

Consequently, we aimed to elucidate the unique protein profiles after KPE in patients with BA using an innovative overlapping window data-independent acquisition-based proteome analysis to better understand profiles specifically related to host-derived proteins in stools.

## Materials and Methods

### Study Design and Subjects

Four individuals with well-controlled conditions (Group A), 4 individuals with repeated cholangitis (Group B), and 3 individuals with prolonged jaundice without cholangitis (Group C), all of whom underwent KPE, were included in the stool proteome analysis. Additionally, 15 healthy individuals without BA (Group D) were recruited for comparison (Table). The stools, once defecated, were immediately preserved at −80 °C.

**Table:** Clinical Features of Participants in This Study

Condition	Group	Title	Sample	Month	Sex	Type of BA	AST	ALT	T-Bil	D-Bil	GGTP	CRP	Number of cholangitis
Well-control	A	1	B1	56	F	Ⅰ-b2-ν	24	9	0.3	0.1	13	0.3	2
		2	B2	51	M	Ⅲ-b1-ν	38	16	0.4	<0.1	36	0.0	0
		3	B3	80	F	Ⅲ	39	32	0.5	<0.1	108	0.1	2
		4	B5	27	F	Ⅲ-c1-ν	127	102	0.2	0.1	53	0.1	0
		Ave.		54			57	40	0.4	0.1	53	0.1	1
LDLT	B	1	L2	8	F	Ⅲ-a1-ν	288	179	10.4	7.3	324	1.8	3
		2	L3	7	F	Ⅲ-b1-ν	277	105	25.5	15.3	66	0.8	5
		3	L4	7	M	Ⅲ-c1-ν	114	73	1.9	1.4	673	0.1	5
		4	L8	9	F	Ⅲ-c1-ν	36	21	4.9	3.7	353	5.7	3
		Ave.		8			179	95	10.7	6.9	354	2.1	4
	C	1	L5	16	F	Ⅲ-d-μ	189	196	2.4	1.7	903	0.2	0
		2	L6	7	F	Ⅲ-b1-ν	178	96	4.1	3.1	188	0.2	0
		3	L7	5	M	Ⅲ-b2-o	362	204	19.4	13.6	338	0.1	0
		Ave.		9			243	165	8.6	6.1	476	0.2	0

	Group		Sample	Month	Sex	Non-BA	AST	ALT	T-Bil	D-Bil	GGTP	CRP	Number of cholangitis
Healthy	D	1	H2	90	M	IH	26	14	0.7	–	11	<0.02	0
		2	H3	57	F	Hr	34	12	0.4	–	14	0.2	0
		3	H4	59	M	UDT	27	9	0.4	–	8	0.1	0
		4	H5	31	M	Hy	29	12	0.4	–	–	0.0	0
		5	H6	42	M	Hy	32	14	0.5	–	–	0.1	0
		6	H7	56	M	IH	29	12	0.4	<0.1	–	<0.02	0
		7	H8	29	M	IH	27	12	0.3	–	–	0.1	0
		8	H9	57	F	IH	26	9	0.8	–	–	<0.02	0
		9	H10	41	F	IH	30	13	0.3	–	12	<0.02	0
		10	H11	16	M	UDT	36	18	0.4	–	–	0.0	0
		11	H12	12	M	UH	48	21	0.5	–	–	<0.02	0
		12	H13	12	M	UDT	39	15	0.5	–	–	<0.02	0
		13	H14	23	M	UDT	43	39	0.3	–	–	0.0	0
		14	H15	12	M	UDT	39	21	0.4	–	10	0.0	0
		15	H16	35	F	UH	33	17	0.6	–	14	0.0	0
		Ave.		38			33	16	0.5		12	–	0

ALT, alanine aminotransferase (U/L); AST, aspartate aminotransferase (U/L); D-Bil, direct bilirubin (mg/dL); GGTP, gamma-glutamyl transpeptidase (U/L); Hr, Hirschsprung disease after curative surgery; Hy, hydrocele; IH, inguinal hernia; T-Bil, total bilirubin (mg/dL); UDT, undescended testicle; UH, umbilical hernia.

### Proteome Analysis

Pretreatment for shotgun proteome analysis was performed as described previously.^8^ Proteomic analysis of the stool samples was conducted according to previously established protocols.^6^^,^^7^ In brief, soluble proteins within the stool samples, prepared in Tris-buffered saline with Tween 20 and protease inhibitors, were extracted by pipetting and inversion after a 30-minute incubation on ice. After centrifugation to remove insoluble matter, the supernatants were transferred to new tubes and subjected to trichloroacetic acid precipitation (final concentration, 12.5% v/v), followed by washing with acetone and drying using an open lid. The dried samples were redissolved in 100 mM Tris-HCl (pH 8.0), 4% sodium dodecyl sulfate, and 20 mM NaCl using a water bath sonicator (Bioruptor UCD-200, SonicBio Corporation, Kanagawa, Japan). The digested peptides were analyzed via data-independent acquisition-based mass spectrometry using an UltiMate 3000 RSLCnano system (Thermo Fisher Scientific) coupled to an Orbitrap Exploris 480 mass spectrometer (Thermo Fisher Scientific) with an InSpIon system.^9^ MS1 spectra were collected in the range of *m/z* 495–745, at a resolution of 15,000, with an automatic gain control target of 3 × 10^6^ and the maximum injection time set to “auto.” MS2 spectra were collected in the range of *m/z* 200–1800 m/z, at a resolution of 45,000, with an automatic gain control target of 3 × 10^6^, the maximum injection time set to “auto,” and normalized collision energy of 26%. The isolation width for MS2 was set to 4 Th. An optimized window arrangement was employed using Scaffold DIA v. 3.2.0 (Proteome Software, Inc, Portland, OR) for the m/z 500–740 window pattern. Subsequently, the DIA-MS data were compared against an in silico human spectral library using data-independent acquisition-mass spectrometry (DIA-NN) v. 1.8.1 (https://github.com/vdemichev/DiaNN).^10^ The spectral library was generated using the human protein sequence database (UniProt ID UP000005640, downloaded in March 2023) using DIA-NN and considering trypsin as the digestion enzyme, allowing for one missed cleavage, a peptide length range of 7–45 bp, a precursor charge ranging from 2–4, a precursor m/z ranging from 490–750, and a fragment ion m/z ranging from 200–1800. Additionally, “FASTA digest for library-free search/library generation,” “deep learning-based spectra, retention times, and ion mobility prediction,” “n-term M excision,” and “C carbamidomethylation” were enabled. The following parameters were applied for the DIA-NN search: a mass accuracy of 10 ppm, MS1 accuracy of 10 ppm, protein inference based on genes, utilization of neural network classifiers in single-pass mode, quantification strategy using robust LC (high precision), cross-run normalization set to “off.” Furthermore, “unrelated runs,” “use isotopologues,” “heuristic protein inference,” and “no shared spectra,” “match-between-run” were enabled. The threshold for protein identification was set at ≤1% for both the precursor and protein false discovery rates.

### Statistical Analysis

Missing values for undetected proteins were imputed using a normal distribution, with the median shifted toward lower abundance (down-shift of 1.8 and width of 0.3) from the median of the measured data distribution of the detected proteins.^11^ The protein abundance levels were log 10-transformed. Principal component analysis (PCA) was conducted using the prcomp function in R (v4.4.2). PCA scores were integrated with clinical metadata to assess their relationship with sex, age, group classification, and aspartate aminotransferase levels. Statistical significance of groupwise differences in PCA space was evaluated using PERMANOVA (adonis2 function in the vegan package, v2.6.10) with Euclidean distances. Effect sizes were calculated for each variable using marginal effects (by = “margin”). Differential abundance analysis between groups was performed using the limma package (v3.62.2),^12^ adjusting for age (months) and sex as covariates. A linear model was fitted to the normalized protein abundance data, followed by empirical Bayes moderation to improve variance estimation. Pairwise comparisons were conducted between groups, and proteins with *P* < .05 were considered statistically significant. Volcano plots were visualized using the ggplot2 package (v3.5.1), with the most significantly upregulated and downregulated proteins being annotated. Gene set enrichment analysis (GSEA) was performed using the gseGO function of the clusterProfiler package (v4.14.4) with the fgsea method.^13^ Proteins were ranked by their signed −log_10_ (*P* value), incorporating fold-change directionality. Enrichment analysis was performed for Gene Ontology Biological Process terms, applying a Benjamini–Hochberg-adjusted *P* value cutoff of .05. The code used in this paper can be obtained from GitHub (https://github.com/my0916/BiliaryAtresia).

## Results

Our proteomic analysis identified 2543 host-derived proteins in stool samples, among which 1458 proteins were detected in at least 10 samples and were used for subsequent analyses (Figure A1), based on our previous study.^7^

### PCA of Stool Proteome

**Figure 1:**
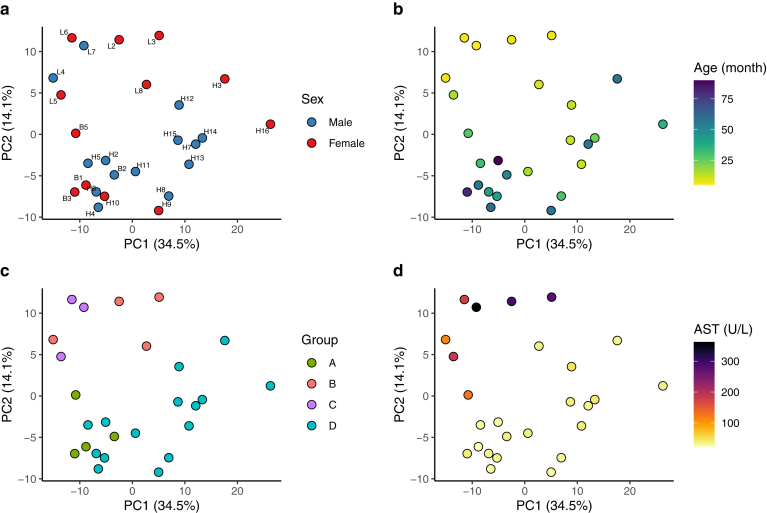
PCA of proteomic data from 26 samples. (A) Colored by sex. (B) Colored by age. (C) Colored by group. (D) Colored by AST levels. AST, aspartate aminotransferase (U/L).

PCA was performed to explore patterns in stool protein composition in this cohort and found no significant differences between male and female stool samples (*P* = .21, Figure 1A) or across different age groups (*P* = .16, Figure 1B). In contrast, significant differences were observed among the groups (*P* = .006). The stool protein profile of the favorable post-KPE Group A (Group A) was similar to that of the healthy control group (Group D), whereas the unfavorable post-KPE groups (Groups B and C) displayed distinct patterns compared to both favorable groups (Groups A and D) (Figure 1C). In addition, the PCA distribution closely mirrored the serum aspartate aminotransferase level distribution (Figure 1D). This indicates that the composition of the stool proteome is influenced more by the patient's condition post-KPE rather than by sex or age.

### Comparative Proteome Analysis: Post-KPE vs Healthy Control

**Figure 2:**
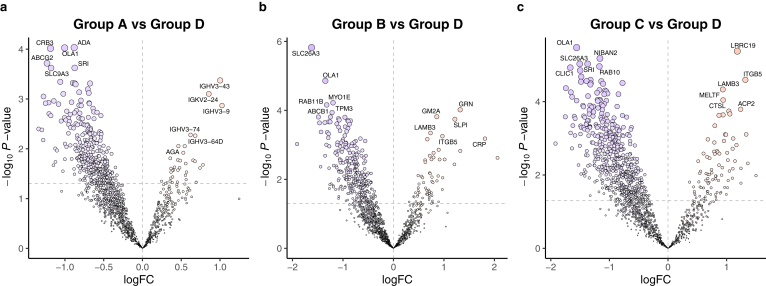
Differential composition of host-derived stool proteins between each post-KPE group and the healthy control group. Volcano plots showing changes in the stool proteome compared to the healthy control group: (A) favorable group, (B) repeated cholangitis, (C) prolonged jaundice. The x-axis represents the effect size, indicated by logFC, while the y-axis displays the statistical significance, represented by the −log_10_*P* value. Horizontal dotted lines indicate *P* = .05. A positive logFC indicated a higher abundance in the post-KPE group, whereas a negative logFC indicated a lower abundance. The 6 most significantly upregulated (positive logFC) and downregulated (negative logFC) proteins were labeled in each plot. logFC, log_2_ fold change; PC, principal component.

We conducted a comparative analysis using previously established methods to explore the unique protein profile in patients following KPE.^7^ Compared to the healthy control group (Group D), each patient exhibited a distinct pattern, as illustrated in Figure 2. Notably, immunoglobulin proteins such as immunoglobulin heavy variable 3–43 (IGHV3–43), kappa variable 2–24 (IGKV2–24), heavy variable 3–9 (IGHV3–9), heavy variable 3–74 (IGHV3–74), and heavy variable 3–64D (IGHV3–64D) were abundant, whereas adenosine deaminase (ADA), obg-like ATPase 1 (OLA1), protein crumbs homolog 3 (CRB3), broad substrate specificity ATP-binding cassette transporter ABCG2 (ABCG2), sorcin (SRI), and sodium/hydrogen exchanger 3 (SLC9A3) were less abundant in the favorable group (Group A) (Figure 2A). Even when comparing healthy controls pre- and post-KPE, the stool protein levels were significantly different post-KPE, indicating that the native liver function of BA following KPE differed from that of healthy individuals. The repeated cholangitis group (Group B) exhibited an abundance of progranulin (GRN), ganglioside GM2 activator (GM2A), antileukoproteinase (SLPI), laminin subunit beta-3 (LAMB3), integrin beta-5 (ITGB5), and C-reactive protein (CRP), and lower expression of chloride anion exchanger (SLC26A3), OLA1, unconventional myosin-Ie (MYO1E), ras-related protein Rab-11B (RAB11B), tropomyosin alpha-3 chain (TPM3), and ATP-dependent translocase ABCB1 (ABCB1) (Figure 2B). Notably, CRP, an inflammatory marker primarily produced by hepatocytes,^14^ was elevated in the stool of individuals with recurrent cholangitis. This suggests that stool proteins may serve as a reservoir for inflammatory substances originating from the liver. Additionally, leucine-rich repeat-containing protein 19 (LRRC19), ITGB5, LAMB3, melanotransferrin (MELTF), lysosomal acid phosphatase (ACP2), and procathepsin L (CTSL) were more abundant, and OLA1, protein Niban 2 (NIBAN2), SLC26A3, SRI, ras-related protein Rab-10 (RAB10), and chloride intracellular channel protein 1 (CLIC1) were less abundant in the prolonged jaundice group (Group C) (Figure 2C). Particularly, OLA1 levels were reduced in the post-KPE group, while SLC26A3 levels were decreased and LAMB3 levels were increased in both unfavorable groups (Groups B and C). OLA1 is a member of the Obg family and the YchF subfamily of P-loop GTPases, which regulate various cellular processes including stress responses,^15^ and works as an intrinsic stress response regulator such as oxidative stress^16^ and heat shock.^17^ This suggests that the post-KPE group may exhibit enhanced physiological responses to stress compared to healthy controls.

### Comparative Proteome Analysis: Unfavorable vs Favorable Post-KPE

**Figure 3:**
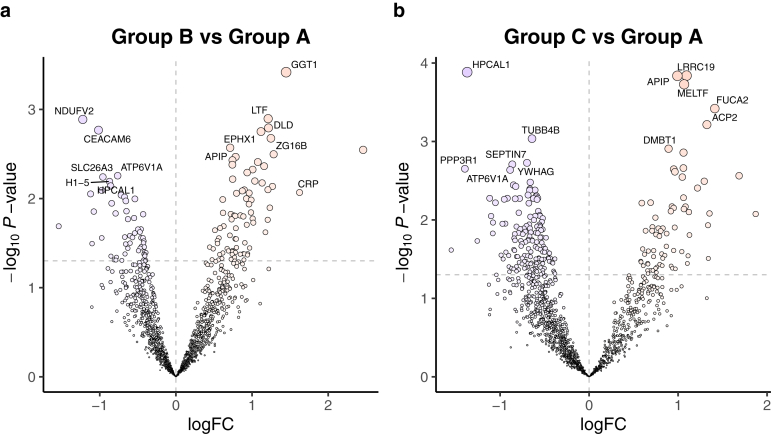
Differential composition of host-derived stool proteins between favorable group and unfavorable group post-KPE groups. Volcano plots showing changes in the stool proteome compared to the favorable group: (A) repeated cholangitis and (B) prolonged jaundice. The x-axis represents the effect size, indicated by logFC, while the y-axis displays the statistical significance, represented by the −log_10_*P* value. Horizontal dotted lines indicate *P* = .05. A positive logFC indicates higher abundance in the unfavorable group, whereas a negative logFC indicates lower abundance. The 6 most significantly upregulated (positive logFC) and downregulated (negative logFC) proteins were labeled in each plot. CRP is specifically labeled in Figure 4A. logFC, log_2_ fold change.

We conducted a comparative analysis to explore the unique protein profiles of the unfavorable groups following KPE. Among the post-KPE groups in the comparative analysis with the favorable group (Group A), glutathione hydrolase 1 proenzyme (GGT1), lactotransferrin (LTF), dihydrolipoyl dehydrogenase (DLD), epoxide hydrolase 1 (EPHX1), zymogen granule protein 16 homolog B (ZG16B), and methylthioribulose-1-phosphate dehydratase (APIP) were more abundant, whereas NADH dehydrogenase [ubiquinone] flavoprotein 2 (NDUFV2), carcinoembryonic antigen-related cell adhesion molecule 6 (CEACAM6), V-type proton ATPase catalytic subunit A (ATP6V1A), SLC26A3, histone H1.5 (H1-5), and hippocalcin-like protein 1 (HPCAL1) were less abundant in the group with repeated cholangitis (Group B) (Figure 3A). Additionally, CRP levels were elevated in the stools of patients with recurrent cholangitis (Figure 3A). Whereas in the prolonged jaundice group (Group C), LRRC19, APIP, MELTF, plasma alpha-L-fucosidase (FUCA2), ACP2, and deleted in malignant brain tumors 1 protein (DMBT1) were more abundant, whereas HPCAL1, tubulin beta-4B chain (TUBB4B), 14-3-3 protein gamma (YWHAG), septin-7 (SEPTIN7), calcineurin subunit B type 1 (PPP3R1), and ATP6V1A were less abundant (Figure 3B). Moreover, APIP levels were increased, whereas HPCAL1 and ATP6V1A levels decreased in both groups. HPCAL1 is known to inhibit the growth of hepatocellular carcinoma cells by regulating cell cycle progression.^18^ The development of malignant hepatic tumors is also recognized as a long-term complication following KPE.^1^ Therefore, the reduced abundance of this protein in both unfavorable groups may reflect the progression of these malignancies.

### Analysis of Biological Processes Enriched in Unfavorable Groups

**Figure 4:**
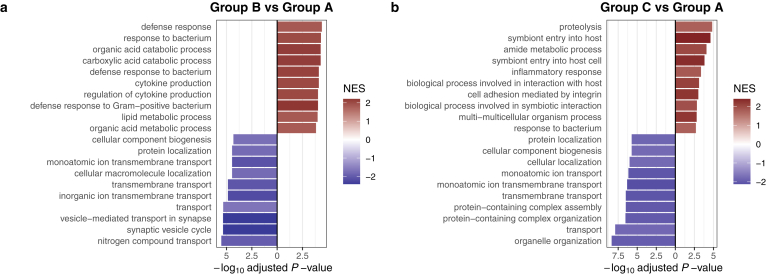
GSEA: Top 10 GO terms of the unfavorable groups (Group B and Group C). Bar plots showing the top 10 significantly enriched GO terms of proteins that were abundant in the unfavorable (red) and favorable groups (blue): (A) repeated cholangitis and (B) prolonged jaundice. *P* values were calculated using a permutation test with Benjamini–Hochberg correction based on the GSEA algorithm implemented in fgsea. GO, Gene Ontology; NES, normalized enrichment score.

GSEA was performed to explore the biological processes enriched in the unfavorable group. Group B (repeated cholangitis) exhibited enrichment in defense response, response to bacterium, organic acid catabolic process, carboxylic acid catabolic process, defense response to bacterium, cytokine production, regulation to cytokine production, defense response to gram-positive bacterium, lipid metabolic process, and organic acid component biogenesis (Figure 4A). Conversely, Group C demonstrated enrichment in proteolysis, symbiont entry into host, amide metabolic process, symbiont entry into host cell, inflammatory response, biological process involved in interaction with host, cell adhesion mediated by integrin, biological process involved in symbiotic interaction, multi-multicellular organism process, and response to bacterium, which were the top 10 enriched Gene Ontology terms (Figure 4B). In summary, biological processes related to the defense response to bacteria were enriched in the group with repeated cholangitis.

## Discussion

**Figure 5:**
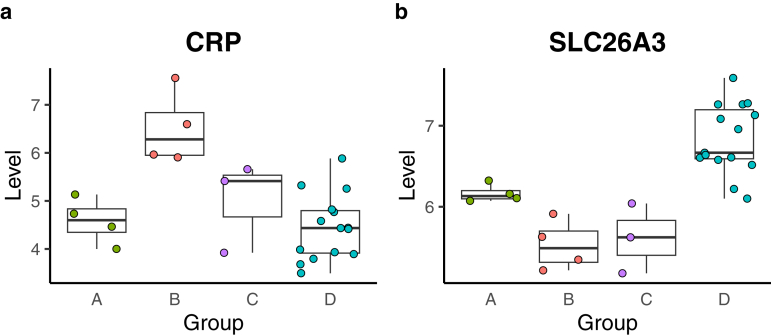
Comparison of stool protein levels detected by proteome analysis across groups, (A) CRP and (B) SLC26A3. Each box represents a different group: Group A for favorable outcomes, Group B for repeated cholangitis, Group C for prolonged jaundice following KPE, and Group D for healthy controls.

In this study, we aimed to elucidate the post-KPE intestinal environment by examining host-derived proteins in the stools of patients with BA. We identified 2543 host-derived proteins in the stool samples using data-independent acquisition mass spectrometry for deep proteome analysis. Focusing on the 1458 proteins detected in at least 10 samples (Figure A1) and applying an imputation method as described in our previous study,^7^ we identified distinct protein variations in the stools of patients post-KPE compared to healthy controls (Figure 2). Particularly, significantly different host-derived protein profiles were observed in patients with poor prognosis post-KPE in the KPE group (groups B and C) (Figure 3). Notably, CRP levels were elevated in the stools of patients with repeated cholangitis (Group B) (Figure 5A). CRP is typically produced by hepatocytes in the liver^14^; therefore, it may be continuously excreted into the digestive system via bile flow, even when serum CRP levels are not significantly elevated (Table, B2, and B3). This finding suggests that stool CRP levels could serve as a potential biomarker for repeated cholangitis, which could assist in decision-making for subsequent liver transplantation following KPE. Additionally, SLC26A3, which is a crucial Cl^–^/HCO3^–^ exchanger located in the apical membrane of intestinal epithelial cells, plays a key role in regulating fluid and electrolyte absorption in the intestine^19^ and was reduced in the unfavorable groups (Groups B and C) (Figure 5B). SLC26A3 expression is notably decreased in animal models of infectious colitis and inflammation-associated diarrhea,^20^ with genome-wide association studies in Japanese populations indicating that SLC26A3 deficiency is associated with an increased risk of developing ulcerative colitis.^21^ Therefore, a reduction in SLC26A3 levels in the stools of the unfavorable group potentially indicated poor intestinal conditions, which were associated with compromised liver function following KPE. Furthermore, GSEA highlighted the involvement of proteins associated with immune responses to bacterium in the repeated cholangitis group (Group B) (Figure 4A). These results possibly reflect a shift in the intestinal environment in repeated cholangitis post-KPE, inducing the host immune response to maintain gut homeostasis.

While this study offers valuable insights, it has limitations, such as a small sample size and age disparities between groups, hindering the intended comparisons. However, PCA analysis revealed significant differences between the groups (*P* = .006, Figure 1C), while no statistically significant differences were observed in stool protein composition across age groups (*P* = .16, Figure 1B). These findings suggest that stool protein composition is more strongly influenced by a patient’s condition than by age. Additionally, although we did not explore the correlation between the microbiome and proteome in the stools of the patients, previous studies have suggested that the gut microbiome changes post-KPE owing to alterations in bile flow. Collectively, these findings warrant further investigation of stool proteomes related to bacterial composition before and after KPE. Nevertheless, conducting a comprehensive proteomic analysis of stools holds promise as a novel approach for elucidating the intestinal environment in patients with BA post-KPE. Given the scarcity of methods to evaluate gastrointestinal conditions, validating our findings in larger cohorts is essential to ensure their clinical relevance.

## Conclusion

This study reports an investigation of stool protein profiles in patients with BA after KPE. Distinct findings were identified regarding the protein profiles of stools post-KPE, particularly in patients with poor clinical outcomes. Thus, our results suggest that proteomic analysis of stool samples has the potential to depict the intestinal environment of patients with BA after KPE, which is influenced by their clinical course. Consequently, examining these stool proteins, as identified through proteomic analysis, provides an opportunity to elucidate the gastrointestinal conditions of postoperative BA, opening new avenues for clinical approaches. Furthermore, it may not only assist in evaluating gastrointestinal conditions but also serve as a means to assess the status of the native liver following KPE.

## Supplementary Materials

Figure A1
