# Supplementary material for: Assessing Native Liver Post-Kasai Portoenterostomy for Biliary Atresia Through Stool Proteome Analysis
Source: Gastro Hep Adv. 2025 Apr 29;4(8):100688. doi: 10.1016/j.gastha.2025.100688 (PMC12205798; doi:10.1016/j.gastha.2025.100688)
Supplement: Figure A1 [file mmc1.pdf]

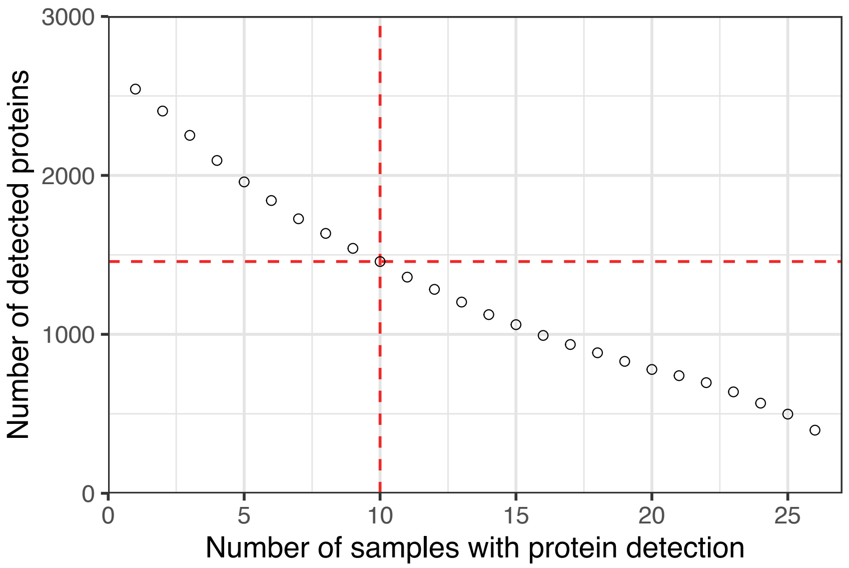

Supplementary Fig.1 Detection of human-derived stool proteins using proteomic analysis

The x-axis represents the number of samples in which the protein was detected.
